# Double-grating monochromatic beamline with ultrafast response for FLASH2 at DESY

**DOI:** 10.1107/S1600577517013777

**Published:** 2018-01-01

**Authors:** Luca Poletto, Fabio Frassetto, Günter Brenner, Marion Kuhlmann, Elke Plönjes

**Affiliations:** a National Research Council – Institute of Photonics and Nanotechnologies, Via Trasea 7, Padova 35136, Italy; b Deutsches Elektronen Synchrotron – DESY, Notkestraße 85, Hamburg 22603, Germany

**Keywords:** time-delay-compensated monochromator, diffraction, variable-line-spaced grating, free-electron laser

## Abstract

The preliminary design of a monochromatic beamline for FLASH2 at DESY is presented. The monochromator is tunable in the 50–1000 eV energy range with resolving power higher than 1000 and temporal response below 50 fs over the whole energy range. A time-delay-compensated configuration using the variable-line-spacing monochromator design with two gratings is adopted: the first grating disperses the radiation on its output plane, where the intermediate slit performs the spectral selection; the second grating compensates for the pulse-front tilt and for the spectral dispersion due to diffraction from the first grating.

## Abstract 2

The preliminary design of a monochromatic beamline for FLASH2 at DESY is discussed.

## 1. Introduction   

Free-electron laser (FEL) sources provide extreme-ultraviolet (XUV) and X-ray radiation with ultrashort time duration, high spatial coherence and an increase of six to eight orders of magnitude on the peak brilliance with respect to synchrotron radiation sources (Huang & Kim, 2007 ▸). These characteristics make FEL sources useful for a wide range of applications, including atomic and molecular physics, ultrafast X-ray science, advanced material studies, ultrafast chemical dynamics, biology and medicine (Yabashi & Tanaka, 2017 ▸). There are several operating FEL facilities already open to users’ experiments: FLASH in Germany (Ackermann *et al.*, 2007 ▸), SACLA-XFEL in Japan (Ishikawa *et al.*, 2012 ▸), LCLS in USA (Emma *et al.*, 2010 ▸), FERMI in Italy (Allaria *et al.*, 2012 ▸) and the incoming European XFEL in Germany (Abela *et al.*, 2006 ▸) and SwissFEL in Switzerland (Ganter, 2010 ▸).

The handling and conditioning of ultrashort coherent FEL pulses has required the development of suitable optical technologies (Canova & Poletto, 2015 ▸). In particular, this paper is focused on the monochromatization of FEL pulses to go beyond the intrinsic resolution of self-amplified spontaneous emission (SASE) FELs. Grating monochromators are already used at FLASH (Martins *et al.*, 2006 ▸; Gerasimova *et al.*, 2011 ▸) and LCLS (Schlotter *et al.*, 2012 ▸).

The use of gratings to realise XUV monochromators with ultrafast time response is well established for high-order laser harmonics, where the problem of pulse length preservation has been extensively studied (Poletto *et al.*, 2012 ▸). Both the single- and the double-grating design are used. In the first case, when using a single grating, a residual pulse-front tilt due to diffraction has to be accepted at the output of the monochromator, that can however be minimized by choosing a suitable geometry to obtain temporal responses in the range of a few tens of femtoseconds in the XUV (Frassetto *et al.*, 2011 ▸). In the second case, two consecutive gratings are employed: the first one performs the spectral selection on an intermediate slit while the second one corrects for the pulse-front tilt introduced by the diffraction. Double-grating instruments have already been demonstrated to give time resolution below 10 fs in the XUV (Poletto *et al.*, 2009 ▸; Ito *et al.*, 2010 ▸; Igarashi *et al.*, 2012 ▸).

In this paper, we present the preliminary design of a monochromator beamline for FLASH2 at DESY (Faatz *et al.*, 2016 ▸; Plönjes *et al.*, 2016 ▸). The monochromator is designed for the 50–1000 eV energy range, *i.e.* 1.2–25 nm wavelength range, with resolving power λ/Δλ higher than 1000 and temporal response below 50 fs over the whole energy range, *i.e.* a temporal elongation of the initial FEL pulse of below 50 fs. The optical design discussed here originates from the variable-line-spaced (VLS) grating monochromator that is already used at LCLS (Heimann *et al.*, 2011 ▸). Different from the conditions at LCLS, where the energy range of the monochromator is 500–2000 eV and the pulse-front tilt given by the grating is below 30 fs, FLASH2 is operated at lower energies and thus the stretching given by the single-grating configuration would be unacceptable, as discussed later. Therefore, a second grating is added to realise a time-delay-compensating configuration that corrects for the pulse-front tilt to below 10 fs.

The paper is organized as follows: the single-grating configuration is initially discussed to show the limitations on the temporal duration of the output monochromatic pulse; then the double-grating configuration is presented and its performance is discussed in detail.

## 2. Single-grating monochromator for ultrafast pulses   

A single grating performs the spectral selection in the simplest optical configuration; however, a residual pulse-front tilt has to be tolerated at the output. Indeed, each ray that is diffracted by two adjacent grooves is delayed by *m*λ, where λ is the wavelength and *m* is the diffracted order. The total pulse-front tilt is |*m*|λ*N*, where *N* is the number of illuminated grooves. Therefore, the pulse stretching depends on the illuminated area on the grating.

Once the resolving power, *R* = λ/Δλ, has been defined, the Rayleigh criterion states that the minimum number of grooves *N*_min_ that have to be involved in the diffraction to support such a resolving power is |*m*|*N*_min_ = λ/Δλ, where Δλ is the half-height spectral width. The corresponding half-width variation of the optical paths at the grating output is ΔOP_min_ ≃ (1/2)|*m*|λ*N*_min_ = (1/2)λ^2^/Δλ. It follows that the diffraction from a grating gives a lower limit for the pulse-front tilt Δτ_G,min_ given by
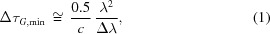
where *c* is the speed of light in a vacuum. This value is quite close to the Fourier limit, which states that the minimum pulse duration Δτ for a given bandwidth is
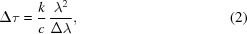
where *k* depends on the pulse shape, *e.g.* *k* ≃ 0.44 for a Gaussian pulse and *k* ≃ 0.32 for a sech^2^-shaped pulse. Therefore the single-grating design can be adopted for the monochromatization of ultrashort pulses without altering in a significant way the pulse duration beyond the Fourier limit, provided that the number of illuminated grooves times the diffracted order is equal to the actual resolving power (Poletto & Frassetto, 2010 ▸). The aim of the optical design is to find the parameters that fulfil the requirements on spectral range, spectral resolution, efficiency, *etc*. and to minimize at the same time the illuminated area on the grating to reduce the pulse-front tilt. Unfortunately, fulfilling of the latter condition is not trivial, since the illuminated area depends on the source divergence, that is assigned, and geometrical parameters, such as the distance from the source and the incident angle, that often are subject to constraints.

**Figure 1:**
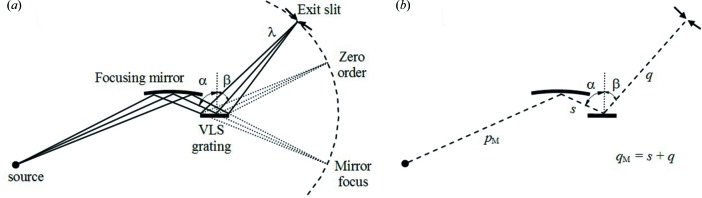
(*a*) Schematic layout of the VLS grating monochromator. (*b*) Definition of parameters: *p*_M_ is the entrance arm of the mirror; *s* is the mirror-to-grating distance; *q* is the grating-to-slit distance; α and β are the incidence and diffraction angles, respectively.

In the following we will concentrate on a VLS grating monochromator. It was proposed by Hettrick & Bowyer (1983 ▸) and used for synchrotron radiation beamlines (Underwood & Koch, 1997 ▸) and high-order laser harmonics (Poletto *et al.*, 2003 ▸). Recently, the VLS design has been adopted also for the monochromatic beamline at LCLS (Heimann *et al.*, 2011 ▸). The optical layout is shown in Fig. 1 ▸. A flat VLS grating is illuminated by the light converging from a focusing mirror and diffracts the radiation onto the exit slit. The wavelength scanning is performed by rotating the grating around an axis passing through its centre to change the incidence angle α at constant subtended angle, *i.e.* α + β = constant. The variable groove spacing provides the additional free parameters to keep the focus on the slit plane at the different wavelengths and to correct for high-order aberrations, namely coma and spherical aberration. The design is rather simple from the opto-mechanical point of view, because only two optical elements are required and the wavelength is scanned by a single rotation.

The incident angle is changed following the equation
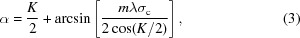
where *K* is the subtended angle: *K* = α + β, α and β are the incidence and diffracted angles, respectively, and σ_c_ is the central groove density.

The half-width bandwidth at the output slit, having width *W*, is
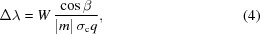
where *q* is the grating arm, that is the distance between the grating centre and the slit.

Let *D* indicate the half-divergence of the source. After the reflection from the concave mirror, the divergence is modified as *D* *p*_M_/(*s* + *q*), where *p*_M_ is the entrance arm of the mirror, *s* is the mirror-to-grating distance and *q* is the grating-to-slit distance. Therefore, the number of grooves illuminated on the grating is *N* = 2*D*[*p*_M_/(*s* + *q*)](σ_c_ *q*)/cosα while the corresponding pulse-front tilt at half-maximum is
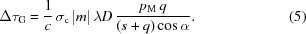
Starting from the required bandwidth Δλ, the parameters *K*, σ_c_ and *q* are chosen to fulfil equation (4) with a given width of the output slit, that is typically *W* ≃ 100 µm. Since *p*_M_, *i.e.* the distance between the source and the mirror, is normally imposed by the geometry of the beamline, the only free parameter to control the pulse-front tilt is the mirror-to-grating distance *s*: the larger the value of *s*, the smaller Δτ_G_.

In the following, we will apply the VLS configuration to the preliminary design of a monochromatic beamline for FLASH2. The main requirements are the following:

(i) Spectral range 4–25 nm (310–50 eV), FLASH2 fundamental emission; 1.2–4 nm (1000–310 eV), FLASH2 harmonics.

(ii) Resolving power λ/Δλ higher than 1000 over the full spectral range.

(iii) Time response below 50 fs (half-width).

When designing a beamline for new-generation FEL sources, there are also some major issues related to the source itself that drive the design:

(i) Due to the high angular and lateral stability of the source, the monochromator works without an entrance slit, *i.e.* the FEL itself acts as the source point.

(ii) Due to high photon flux, horizontal and vertical foci have to be kept separated to reduce the radiation density on the slit blades.

There are also some geometrical constraints to be taken into account for FLASH2:

(i) The first optical element of the beamline, *i.e.* the first deviating mirror, has to be placed 68.9 m away from the source.

(ii) The minimum distance between the first deviating mirror and the grating is 6 m due to space contraints.

(iii) The total length of the beamline from the first deviating mirror can be 23 m at maximum.

The FLASH2 source is assumed to have a size of 200 µm r.m.s. The divergence is taken as 75 µrad r.m.s. at 40 nm and scales as λ^3/4^ (Plönjes *et al.*, 2013 ▸).

### 2.1. Single-grating monochromator for FLASH2   

**Figure 2:**
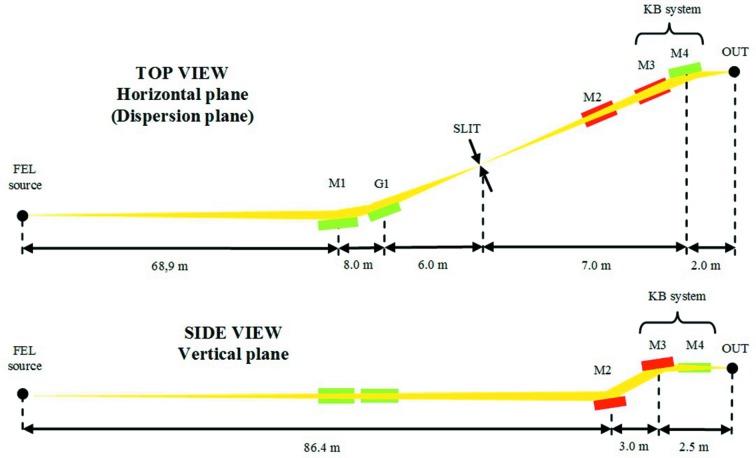
Layout of a monochromatic beamline for FLASH2 with a single grating. The drawing is not to scale.

The single-grating setup is similar to the already existing monochromatic beamline at SLAC. The optical layout is depicted in Fig. 2 ▸. The FEL beam is focused by the plane-elliptical mirror M1 towards the plane VLS grating G1. The latter is illuminated in converging light and diffracts the radiation toward the slit, where the beam is monochromated. The radiation is finally focused to the sample by two plane-elliptical mirrors in the Kirkpatrick–Baez (KB) configuration, M3 and M4. The additional plane mirror M2 is used in combination with the mirror M3 to correct the vertical deviation of the beam and have an output beam parallel to the floor.

The monochromator parameters have been chosen to obtain the required resolving power using a 100 µm slit. They are listed in Table 1 ▸. Two gratings are used to cover the full spectral range. They are used in the first and second orders to optimize the performance in a broad interval. Note that the groove profile has to be blazed, *i.e.* a saw-tooth profile, to have high efficiency at the two diffracted orders. The blaze angle that maximizes the grating efficiency is 1°. The position of the KB focusing stage has been fixed to give a demagnification of 3.5 in the horizontal direction and 36 in the vertical direction, thus giving a half-maximum size of the final focus of about 15 µm × 10 µm.

**Figure 3:**
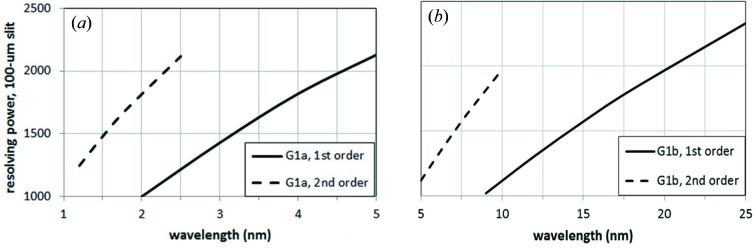
Resolving power on a 100 µm slit of the single-grating monochromator: (*a*) G1a; (*b*) G1b. The data have been calculated using equation (4) and also verified through ray-tracing simulations.

**Figure 4:**
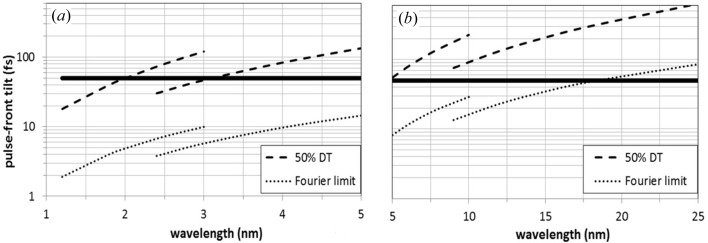
Half-width pulse-front tilt (indicated as 50% DT) of the single-grating monochromator: (*a*) G1a; (*b*) G1b. The Fourier limit for a Gaussian pulse is also shown. The horizontal line indicates the 50 fs value.

The resolving power on a 100 µm slit is shown in Fig. 3 ▸. The use of the first and second orders allows the requirements of using two gratings over the full spectral range to be met. The half-width pulse-front tilt is shown in Fig. 4 ▸. It has been calculated using equation (5) and also verified through ray-tracing simulations using a program explicitly written to calculate the delay of the rays within the beam aperture. Although the temporal stretching has been minimized by increasing the mirror-to-grating distance as required by equation (5), the pulse-front tilt is below 50 fs only for some wavelengths shorter than 3 nm and it becomes as high as 600 fs at 25 nm. Being limited by the pulse-front tilt, the single-grating monochromator cannot be used in any interval within the 3–25 nm range for a temporal resolution below 50 fs. Fig. 4 ▸ shows only the pulse length stretching due to the monochromator, which is added to the initial pulse length of the FEL pulse. A much shorter response is achieved by using a double-grating configuration, as will be discussed in the following section.

## 3. Double-grating monochromator for ultrafast pulses   

Double-grating configurations have been proposed for XUV ultrafast pulses to correct for the pulse-front tilt given by the diffraction from a single grating. In such a configuration the second grating compensates for the temporal stretching and for the spectral spread introduced by the first one. Such a configuration is normally defined as a *time-delay-compensating monochromator*. From the point of view of the ray paths, there are two conditions that the design must comply to: (i) the differences in the path lengths of rays having the same wavelength but with different entrance directions within the beam aperture that are caused by the first grating must be compensated by the second grating, and (ii) two rays at different wavelengths within the spectrum of the pulse to be selected have to be focused on the same point, *i.e.* the global spectral dispersion has to be zero. Both conditions are satisfied by a scheme with two equal concave gratings mounted in a symmetrical way and operated in opposite diffraction orders, *i.e.* the incidence angle on the second grating is equal to the diffraction angle of the first grating. The spectral selection is performed by a slit placed in an intermediate position between the gratings, where the radiation is focused by the first grating. Time-delay-compensating monochromators are almost routinely used for the monochromatization of high-order laser harmonics, as already discussed in the *Introduction* [Sec sec1].

In the following we will concentrate on the double-grating configuration applied to the VLS grating monochromator. The correction of the pulse-front tilt is achieved by inserting a second VLS grating equal to the first one into the optical path after the slit (*i.e.* with the same average groove density and same law for groove space variation), mounted in a symmetrical position with respect to the slit to be illuminated on the same area (*i.e.* both gratings are placed at the same distance from the slit therefore the number of illuminated grooves is the same) and operated in the compensating configuration, that requires to operate the second grating in the opposite diffraction order with respect to the first one and to mount the two gratings in the so-called C configuration (*i.e.* both are faced on the same side).

The condition for time-delay compensation requires that: (i) the same number of grooves is illuminated in both gratings; (ii) the two gratings are used in opposite diffraction order (internal–external or external–internal); (iii) the spectral dispersion is compensated (gratings in C geometry, *i.e.* both faced on the same side). Once these three conditions are fulfilled, the tilt of the pulse-front is corrected. After the second grating, the optical elements can be designed to achieve the desired focus without influencing the pulse-front tilt.

### 3.1. Double-grating monochromator for FLASH2   

**Figure 5:**
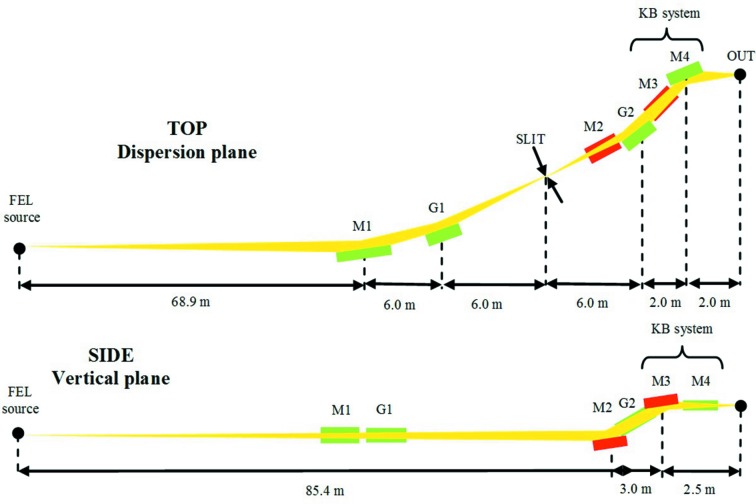
Layout of a monochromatic beamline for FLASH2 with time-delay-compensating configuration. The drawing is not to scale.

The optical layout is shown in Fig. 5 ▸. With respect to the single-grating design, a second grating G2 is added. The distances have been recalculated to fit within the space available for the beamline, in particular the M1-to-G1 distance has been reduced to 6.0 m to reduce the total beamline length within the given constraints.

The monochromator parameters are listed in Table 2 ▸. Two gratings are operated at the first and second orders to cover the full spectral range. The grating subtended angle has been increased to 174° to achieve higher efficiency at short wavelengths. Again, note that the groove profile has to be blazed. The blaze angle that maximizes the grating efficiency is 1°.

**Figure 6:**
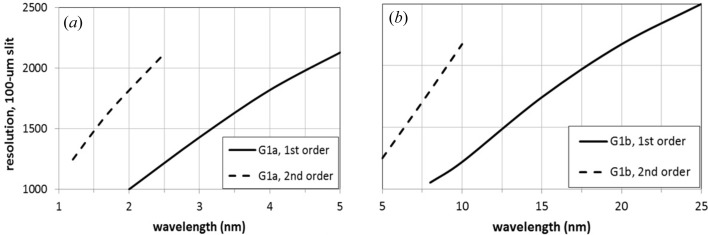
Resolving power on a 100 µm slit of the double-grating monochromator: (*a*) G1a; (*b*) G2a.

The resolving power on a 100 µm slit is shown in Fig. 6 ▸. The use of the first and second orders allows the requirements of using two sets of gratings over the full spectral range to be fulfilled.

#### 3.1.1. Temporal response   

The temporal response is evaluated considering two effects on the ultrafast pulse given by the time-delay-compensating configuration. The first effect is the compensation of the pulse-front tilt, *i.e.* all the rays emitted by the source in different directions at the same wavelength have to travel the same optical path. Ideally the compensation is perfect for a double-grating configuration, although aberrations may give a residual distortion of the pulse-front, which is evaluated through ray-tracing simulations. The second effect is the group delay introduced by the two gratings, *i.e.* different wavelengths within the bandwidth transmitted by the slit travel different paths. Similarly to grating pulse shapers for the visible range, the pair of gratings in the time-delay-compensating configuration can be considered as an XUV pulse shaper, capable of introducing a controllable group delay (Frassetto *et al.*, 2008 ▸; Mero *et al.*, 2011 ▸). In this case the configuration is very asymmetrical, since a large demagnification is used, therefore, a non-negligible group delay is introduced within the bandwidth. The group delay has been calculated by ray-tracing simulations. The resulting optical path decreases linearly with the wavelength and this forces the group delay dispersion to be almost constant and positive.

**Figure 7:**
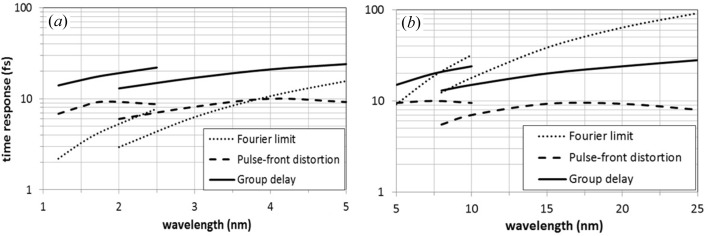
Temporal response of the double-grating monochromator: half-width residual distortion of the pulse-front at the output, group delay and Fourier limit calculated for the half-width bandwidth on a 100 µm slit. (*a*) G1a and G2a; (*b*) G1b and G2b.

The residual distortion of the pulse-front at the output, the group delay and the Fourier limit for the output bandwidth (100 µm slit) are shown in Fig. 7 ▸. The double-grating configuration is very effective in compensating for the pulse-front tilt at the slit, which may be as high as 1 ps at 25 nm, down to a residual sub-10 fs distortion. Fig. 7 ▸ shows only the pulse length distortion due to the monochromator, which is added to the initial pulse length of the FEL pulse. This is the value to be compared with the Fourier limit and with the group delay in order to calculate the ultimate temporal resolution. The results reported in Fig. 7 ▸ show that for wavelengths shorter than 7 nm the group delay is the dominating factor limiting the pulse duration for a Fourier-limited pulse in the 15–25 fs range. For longer wavelengths, the temporal response is dominated by the Fourier limit, which may be as high as 90 fs at 25 nm.

A trade-off between Fourier limit and group delay can be found by acting on the width of the slit. Indeed, the Fourier limit is inversely proportional to the slit width, while the group delay is directly proportional to it. For longer wavelengths, the slit can be opened to ∼200 µm; in such a way the output bandwidth is doubled. Therefore, the Fourier limit will decrease by a factor of two (∼50 fs) and the group delay will increase by the same factor (∼50 fs), giving the best trade-off for temporal resolution at the longer-wavelength side.

It can be concluded that the double-grating configuration will fulfill the requirements on temporal and spectral resolution over the whole interval of operation.

#### 3.1.2. Spot size   

Differently from the single-grating configuration, in the time-delay-compensating configuration the spot size at the output is independent of the slit aperture. This is because the configuration compensates also for the spectral dispersion that occurs on the slit plane, *i.e.* different wavelengths are focused on different points on the slit plane but they are recombined on the same point at the output after the second grating. Therefore, the size of the final image is independent of the width of the slit, since dispersion is compensated. Indeed, the width of the image is the projection of the source size intended as monochromatic that is demagnified by M4 and diffracted by G1. The height of the image is the projection of the source size as demagnified by M3. Given the FLASH2 parameters, the half-width spot size at the output is evaluated by ray-tracing simulations to be 12 µm × 8 µm (H × V). The horizontal (vertical) spot size is here defined as the size of the horizontal (vertical) aperture that transmits 50% of the rays.

#### 3.1.3. Slope errors   

The effects of slope errors on the resolution and on the spot size have been evaluated through ray-tracing simulations. The spectral resolution is affected only by the surface quality of M1 and G1. The decrease of the resolving power is almost negligible even for 2 µrad r.m.s. slope errors on the optics. Indeed, the slope errors limit mainly the spot size at the output, the degradation being almost a factor of two for 2 µrad r.m.s. slope errors. This is the main effect to be considered when defining the desired quality of the optical surfaces. It can be concluded that slope errors below 1 µrad r.m.s. have to be required for all the optical surfaces in order to keep high focusing properties.

#### 3.1.4. Efficiency   

Of particular importance for a beamline design is the total transmission, which depends on the reflectance of the mirrors and on the efficiency of the gratings. With respect to a monochromatic beamline with a single grating, just one optical element has been added to achieve the ultrafast response in the XUV range, namely the second grating. Since the efficiency of a single grating is expected to be in the range 15–35%, the efficiency of the beamline is decreased by a factor of three to eight with respect to the single-grating design. Typically, the higher the energy, the lower the efficiency. For energies below ∼280 eV, where C-coated optics can be used, the total transmission is expected to be in the range 0.1–0.15. For energies in the range 280–650 eV, where Ni-coated optics can be used, the total transmission is expected to be ∼0.05. For energies higher than 280 eV, Pt-coated optics have to be used and the total transmission is expected to be ∼0.01. Furthermore, assuming tangential sizes of 400 mm for the mirrors and 160 mm for the gratings, the vignetting due to the finite sizes of the optics is totally negligible.

The use of the second grating may be a problem, in terms of efficiency, for wavelengths shorter than 4 nm, where the FEL third harmonic is used as a source, since the photon flux is lower than the fundamental by a factor of ∼100 and the grating efficiency is expected to be 20–25% at best. Since the pulse-front tilt of the single-grating configuration is in any case small for short wavelengths (see Fig. 4 ▸), a possible solution would be to use just the first grating for the short wavelengths and take out the second grating. In this case, the focusing KB system and the experimental chamber have to be moved to the new focal position for experiments at short wavelengths: the required rotation is 6° and the resulting (horizontal) translation is ∼20 cm for the KB system and ∼40 cm for the experimental chamber in the proposed design.

## 4. Conclusions   

The preliminary design and expected performance of a monochromatic beamline for FLASH2 have been presented. The beamline operates in the 50–1000 eV energy range and adopts a time-delay-compensating configuration with two VLS gratings to compensate for the pulse-front tilt due to diffraction. Indeed, as shown in the paper, given the characteristics of the FLASH2 source and the proposed requirements on the spectral resolution with a resolving power λ/Δλ higher than 1000, monochromatic ultra-short pulses below 50 fs require the use of two gratings for most of the desired wavelength range. The aim of the design has been to find the best trade-off in terms of optical parameters in order to fulfil the requirements within the whole spectral region of operation.

## Figures and Tables

**Table 1:** Parameters of the VLS grating monochromator for FLASH2, single-grating design

Source-to-M1 distance	68.9 m
M1-to-G1 distance	8.0 m
G1-to-slit distance	6.0 m
Grating subtended angle	172°
Grating G1a	
Interval	1.2–5 nm (1000–250 eV)
Central groove density	600 grooves mm^−1^
Grating G1b	
Interval	5–25 nm (250–50 eV)
Central groove density	150 grooves mm^−1^

**Table 2:** Parameters of the VLS grating monochromator for FLASH2, double-grating design

Source-to-M1 distance	68.9 m
M1-to-G1 distance	6.0 m
G1-to-slit distance	6.0 m
Slit-to-G2 distance	6.0 m
Grating subtended angle	174°
Gratings G1a and G2a	
Interval	1.2–5 nm (1000–250 eV)
Central groove density	600 grooves mm^−1^
Gratings G1b and G2b	
Interval	5–25 nm (250–50 eV)
Central groove density	150 grooves mm^−1^
